# Prevalence of Microbiological and Chemical Contaminants in Private Drinking Water Wells in Maryland, USA

**DOI:** 10.3390/ijerph15081686

**Published:** 2018-08-07

**Authors:** Rianna T. Murray, Rachel E. Rosenberg Goldstein, Elisabeth F. Maring, Daphne G. Pee, Karen Aspinwall, Sacoby M. Wilson, Amy R. Sapkota

**Affiliations:** 1 Maryland Institute for Applied Environmental Health, University of Maryland School of Public Health, 4200 Valley Drive, College Park, MD 20742, USA; rmurray@umd.edu (R.T.M.); rerosenb@umd.edu (R.E.R.G.); swilson2@umd.edu (S.M.W.); 2 Department of Agricultural & Resource Economics, College of Agriculture & Natural Resources, University of Maryland, 2200 Symons Hall, 7998 Regents Drive, College Park, MD 20742, USA; 3 Department of Family Science, University of Maryland School of Public Health, 4200 Valley Drive, College Park, MD 20742, USA; efmaring@umd.edu; 4 University of Maryland Extension, University of Maryland, 2200 Symons Hall, 7998 Regents Drive, College Park, MD 20742, USA; dpee@umd.edu (D.G.P.); Karen@cattailcompany.com (K.A.)

**Keywords:** private wells, groundwater, drinking water, animal feeding operation, fecal coliforms, enterococci, *E. coli*, Maryland

## Abstract

Although many U.S. homes rely on private wells, few studies have investigated the quality of these water sources. This cross-sectional study evaluated private well water quality in Maryland, and explored possible environmental sources that could impact water quality. Well water samples (*n* = 118) were collected in four Maryland counties and were analyzed for microbiological and chemical contaminants. Data from the U.S. Census of Agriculture were used to evaluate associations between the presence of animal feeding operations and well water quality at the zip code level using logistic regression. Overall, 43.2% of tested wells did not meet at least one federal health-based drinking water standard. Total coliforms, fecal coliforms, enterococci, and *Escherichia coli* were detected in 25.4%, 15.3%, 5.1%, and 3.4% of tested wells, respectively. Approximately 26%, 3.4%, and <1% of wells did not meet standards for pH, nitrate-N, and total dissolved solids, respectively. There were no statistically significant associations between the presence of cattle, dairy, broiler, turkey, or aquaculture operations and the detection of fecal indicator bacteria in tested wells. In conclusion, nearly half of tested wells did not meet federal health-based drinking water standards, and additional research is needed to evaluate factors that impact well water quality. However, homeowner education on well water testing and well maintenance could be important for public health.

## 1. Introduction

An estimated 44.5 million people in 13 million households across the United States, 14% of the nation’s population, rely on private domestic wells as their primary drinking water source [1,2]. The Safe Drinking Water Act (SDWA) was originally passed by Congress in 1974 to protect public health by regulating the nation’s public drinking water supply and its sources, including rivers, lakes, reservoirs, springs, and groundwater wells [3]. However, private wells that serve less than 25 people or have less than 15 service connections are neither regulated by the SDWA nor monitored by local regulatory agencies for contaminants that may be associated with adverse human health outcomes [3].

The U.S. Environmental Protection Agency (U.S. EPA) and the National Groundwater Association provide guidance to homeowners and recommend testing private wells annually for a number of parameters including total coliform bacteria, nitrates, total dissolved solids (TDS), and pH [4,5]. As this testing is voluntary, little is known about the level or frequency of testing that is performed by private well owners, or about their knowledge and literacy regarding proper well maintenance, testing, and test results. Data on the microbiological and chemical quality of well water are also scarce. Additionally, many homeowners who utilize private water wells may lack the educational and/or financial resources necessary to address water quality issues associated with private water systems [6,7]. The U.S. Centers for Disease Control and Prevention (CDC) recently reported a significant decrease in the annual proportion of reported waterborne disease outbreaks between 1971 and 2006 in public drinking water systems; however, an increase was observed in the annual proportion of outbreaks associated with individual (private) water systems over the same time period [8]. More recently, a study in North Carolina found that between 2007 and 2013, 99% of emergency department visits for acute gastrointestinal illness caused by microbial contamination of drinking water were associated with private wells [9]. While the CDC report and the North Carolina study suggest a potential public health issue regarding private wells, the lack of information on private well water quality and monitoring makes it difficult to determine the specific contaminants causing these observed illnesses.

Recent studies conducted in Pennsylvania, Virginia, and Wisconsin reported that 40–50% of private wells exceed at least one SDWA health-based standard, most often for coliform bacteria [10,11,12,13,14]. These studies and others have demonstrated the influence of factors such as well construction characteristics, local geology, and climatic conditions on private well water quality [10,12,15,16,17]. Wallender et al. (2014) evaluated data from the CDC’s Waterborne Disease and Outbreak Surveillance System (WBDOSS) and found that improper design, maintenance, or location of private wells and septic systems contributed to 67% of reported outbreaks from groundwater contamination from 1971 and 2008 [18]. In Maryland, approximately 19% of the population relies on private wells [2], however, only one previous study has investigated private well water quality in the state [19]. Additionally, previous studies have indicated that homeowners generally do not regularly test their private wells or seek technical assistance unless they perceive a water quality problem at the point of use [12,20,21], illustrating a need to educate well owners on the importance of monitoring their wells. To address this need, we developed safe drinking water clinics in several Maryland counties. The goals of the clinics were as follows: (1) to educate well owners on proper well maintenance practices and health risks of contaminated wells; (2) to provide well water quality testing in accordance with EPA guidelines; and (3) to characterize the prevalence of microbiological and chemical contaminants in tested wells.

After the clinics were completed, we recognized a need to evaluate potential environmental factors that could influence well water quality in Maryland. Recently, Li et al. (2015) investigated microbiological contamination of domestic and community supply wells in California’s Central Valley, a region with intensive animal agriculture [22]. Approximately 5.9% and 10.3% of wells were positive for generic *E. coli* and *Enterococcus* spp., respectively, with significant associations observed between concentrations of enterococci and proximity of wells to animal feeding operations [22]. In Maryland, there are 12,200 registered farms, including a number of animal feeding operations [23]. In 2014, the state ranked ninth among U.S. states in broiler chicken production [23]. Maryland also has dairy and livestock farms, with 49,000 milk-producing cows and another 190,000 beef cattle and calves [23]. If wells are not properly constructed or maintained, there is potential for surface contaminants from agricultural operations to influence well water quality. As such, we leveraged the well water data collected during the safe drinking water clinics to investigate the possible association between the presence of animal feeding operations and well water quality.

## 2. Materials and Methods

### 2.1. Safe Drinking Water Clinics

**Figure 1:**
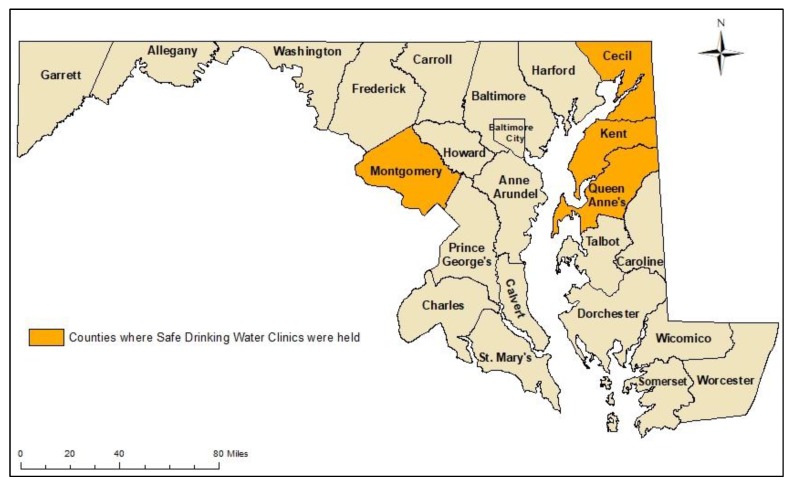
Maryland counties where safe drinking water clinics were held.

Between 2012 and 2014, five safe drinking water clinics were held in four Maryland counties: Cecil (two clinics), Kent, Montgomery, and Queen Anne’s (Figure 1, Table 1). Cecil, Kent, and Queen Anne’s counties are located on Maryland’s Eastern Shore (Figure 1), where a large number of homes rely on private wells. The Eastern Shore is highly agricultural and has the highest concentration of animal feeding operations (particularly broiler chicken operations) in the state [24,25,26]. Montgomery County is also characterized by a large number of homes that rely on private wells; however, there are fewer animal feeding operations in this county.

**Figure 2:**
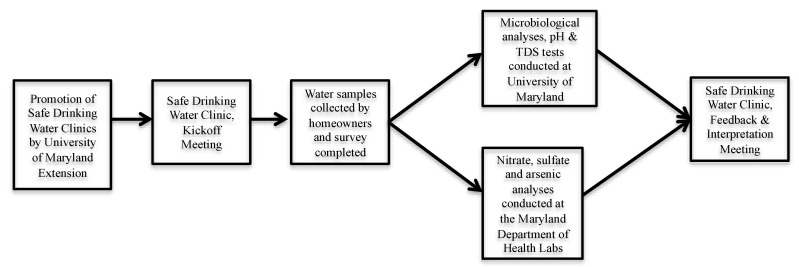
University of Maryland safe drinking water clinic approach. TDS—total dissolved solids.

Clinic participants (*n* = 150) were recruited at county health fairs, farmers’ markets, and through promotional material on community email listservs and local newspapers. Participants were limited to homeowners in the aforementioned counties with private wells who were interested in participating in the clinics. The safe drinking water clinics were a multi-stage process (Figure 2) that began with a kick-off meeting where registered participants were told of the purpose and significance of the project, provided with water sampling instructions and kits (gloves, two 1 L sterile, polypropylene, wide-mouth Nalgene environmental sampling bottles (Nalgene, Lima, OH, USA) and a large Ziploc bag), and taught how to sample their well water from kitchen or bathroom faucets in accordance with standard protocols. A paper-based survey that was developed by our research and extension teams, and approved by the University of Maryland College Park Institutional Review Board, was also given to participants at the kick-off meetings. The survey included questions on well characteristics, homeowner well management practices, prior testing conducted (if any), demographic questions (age, sex, race/ethnicity, and income level), and general health-related questions, including, “In the past month, have you experienced diarrhea?” and “In the past month, have you experienced vomiting?”

Participants returned their water samples and completed surveys to their local University of Maryland (UMD) extension office. Samples were kept on ice and transported to the lab within 12 h. Following completion of laboratory analyses (described below), a second follow-up clinic was held where water quality results were returned to participants who provided water samples. The results were individually and confidentially interpreted for participants and potential solutions for wells that did not meet federal standards were discussed where necessary. A follow-up survey was sent to all participants within 12 months after the clinics were conducted to document actions taken by well owners to solve water quality problems or improve the management of their water supply as a result of attending our clinics (data not shown).

### 2.2. Laboratory Analyses

Water samples were analyzed within 24 h of collection for total coliforms, fecal coliforms, *E. coli*, *Enterococcus* spp., and *Salmonella* spp., according to standard U.S. EPA membrane filtration methods [27,28,29,30]. Briefly, 100 mL of each sample was filtered through 0.45-μm, 47-mm mixed cellulose ester filters. The filters were then placed on the appropriate selective media for each microorganism. Membrane-*Enterococcus* Indoxyl-β-d-Glucoside Agar (mEI) was used for the isolation and enumeration of *Enterococcus* spp.; MI Agar was used for the isolation and enumeration of both total coliforms and *E. coli*; and mFC was used for the isolation and enumeration of fecal coliforms. The mEI plates were incubated at 41 °C for 24 h, mFC plates were incubated at 44.5 °C for 24 h and MI plates were incubated at 37 °C for 24 h. For *Salmonella* detection, membranes were placed in lactose broth, vortexed vigorously for 3 min, and incubated for 24 h at 37 °C. An aliquot of this enrichment was transferred to TT (tetrathionate) broth base, Hajna; incubated at 37 °C for 24 h; plated on XLT4; and incubated at 37 °C for 24 h. Positive and negative controls were used during each test, and plate counts were performed immediately after incubations.

TDS (mg/L) and pH were analyzed using the Pocket Pal TDS Tester and the Stream Survey Test Kit, respectively (Hach Company, Loveland, CO, USA) [31,32]. For nitrate testing, 1 L of each sample was placed into a sterile 1 L polypropylene Nalgene environmental sampling bottle (Nalgene, Lima, OH, USA), 2 mL sulfuric acid solution was added, and the pH was adjusted to <2. For total arsenic testing, 1 L of each sample was placed into a sterile 1 L polypropylene Nalgene environmental sampling bottle (Nalgene, Lima, OH, USA), 2–3 mL of nitric acid solution was added, and the pH was adjusted to <2. The remainder of each water sample was used for sulfate testing. Nitrate and sulfate testing were completed at the Maryland Department of Health (MDH) Labs using an Agilent (Santa Clara, CA, USA) gas chromatograph-mass spectrometer. Nitrate analyses were performed according to U.S. EPA Method 353.2, while sulfate analyses were performed according to U.S. EPA Method 375.2 [33,34]. Total arsenic testing was also completed at the MDH Labs using an Agilent (Santa Clara, CA, USA) inductively-coupled plasma-mass spectrometer per U.S. EPA Method 200.8 [35]. All quality control/quality assurance approaches recommended by the U.S. EPA methods were employed, including analyses of quality control samples, as well as laboratory reagent blanks and fortified blanks [33,34,35].

### 2.3. Animal Feeding Operations Data

We obtained animal feeding operations data from the 2007 U.S. Census of Agriculture, National Agricultural Statistics Service [36]. Specifically, we obtained data on the number of animal feeding operations with sales by zip code for the following animal types: broiler chickens, turkeys, aquaculture, sheep or goats, hogs, dairy cattle, and beef cattle. The 2007 Census was used because it is the most recent U.S. Census of Agriculture that provides data at the zip code level.

### 2.4. Statistical Analyses

We performed descriptive statistics on all well water data. We also linked well water data and animal feeding operation data by zip code and used univariate logistic regression models to evaluate associations between the presence of each type of animal feeding operation and detection of indicator bacteria in well water samples. The presence of total coliform bacteria and fecal coliform bacteria were the dichotomous (presence/absence) outcome variables of interest. All statistical analyses were performed in SAS 9.4 (Cary, NC, USA) [37].

## 3. Results

### 3.1. Characteristics of Safe Drinking Water Clinic Participants

A total of 150 homeowners attended our safe drinking water clinics. However, only 118 participants returned both a water sample and a completed survey (Table 2). Only the 118 participants who returned both a water sample and a completed survey were included in this study’s analyses. The Queen Anne’s County clinic drew the most participants (*n* = 28; 23.1%), followed by the first clinic conducted in Cecil County (*n* = 25; 21.4%). A vast majority of participants were white (87.3%) and most were in the 60–69 age group (33.9%). Participants were also well-educated: 29.7% had obtained a Bachelor’s degree and 39.8% had obtained a graduate degree. At the time of the clinics, a large number of participants had lived at their current residence for at least 10–20 years (39%). Twenty-nine (24.6%) participants indicated that they had never tested their well water quality, and 58 (49.2%) participants had only tested their water once. Approximately 12% and 0% of participants experienced diarrhea and vomiting, respectively, within 30 days prior to completing the survey.

### 3.2. Well Water Quality

**Figure 3:**
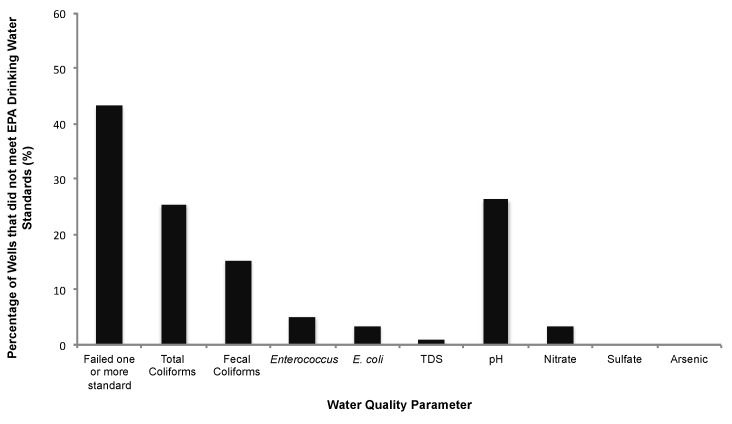
Percentage of tested private wells that did not meet U.S. Environmental Protection Agency (US EPA) drinking water standards.

**Figure 4:**
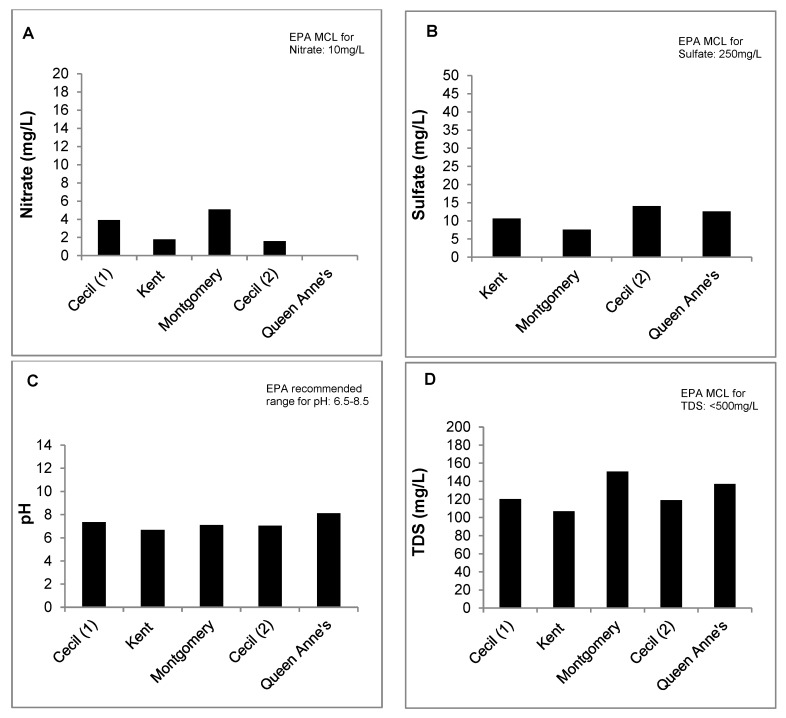
Mean levels of nitrate (Panel **A**), sulfate (Panel **B**), pH (Panel **C**) and total dissolved solids (TDS) (Panel **D**) detected in tested private wells by county [38]. MCL—maximum contaminant level.

Overall, 43.2% of wells tested in this study did not meet at least one EPA health-based drinking water standard (Figure 3). Total coliform bacteria were the most common (25.4%) microbiological contaminant detected. Fecal coliforms (15.3%), Enterococcus spp. (5.1%), and E. coli (3.4%) were also detected. Salmonella was not detected in any of the private wells analyzed in this study. Regarding chemical contaminants, 26% of tested wells did not meet the recommended drinking water standard for pH (Figure 3), with most of these (83.8%) having a pH below the lower limit of 6.5. There were a few wells (16.2%) with a high pH above the recommended limit of 8.5. Nitrate occurred above the 10 mg/L drinking water standard in 3.4% of tested wells, and less than 1% of wells exceeded the recommended limit for total dissolved solids (TDS) of 500 mg/L. None of the wells had an arsenic level above the EPA maximum contaminant level (MCL) for arsenic (10 mg/L). Similarly, none of the wells tested exceeded the EPA MCL for sulfate of 250 mg/L. Although there were individual wells in each county that exceeded the EPA MCLs for some of the chemical water quality parameters investigated, the mean levels in each county were within EPA specifications (Figure 4).

**Figure 5:**
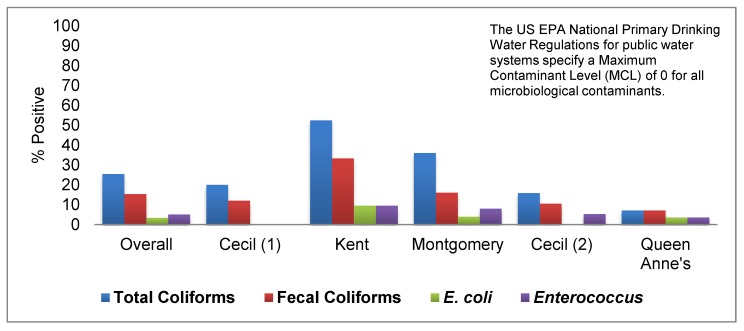
Percentage of tested private wells that were positive for fecal indicator bacteria by county.

Kent County had the highest percentage of wells that tested positive for fecal indicator bacteria, with 52.4% of wells testing positive for at least one type of indicator bacteria (Figure 5). *E. coli* was detected in wells sampled in every county with the exception of Cecil County. *Enterococcus* was detected in samples from all counties; however, it was not detected during the first clinic conducted in Cecil County.

### 3.3. Influence of Animal Feeding Operations on Well Water Quality

Our zip code-level analysis found no evidence that the presence of animal feeding operations influenced the occurrence of fecal indicator bacteria in tested wells (Table 3). In zip codes that contained cattle operations, the contamination of wells by total coliform bacteria was 1.23 times greater than in zip codes that did not contain cattle operations; however, this finding was not statistically significant (Odds Ratio (OR) = 1.23; 95% Confidence Interval (CI) = 0.89, 1.68). In zip codes that contained dairy and aquaculture operations, the contamination of wells by total coliform bacteria was more likely than in zip codes that did not contain one of these operations (dairy operations: OR = 1.12; 95% CI = 0.96, 1.31; aquaculture operations: OR = 1.32; 95% CI = 0.59, 2.93). However, these associations were not statistically significant (Table 3).

Similarly, in zip codes that contained broiler, cattle, dairy, turkey, and aquaculture operations, the contamination of wells by fecal coliform bacteria was more likely than in zip codes that did not contain one of these operations; however, none of these associations were significant. The presence of broiler, hog, and turkey operations in zip codes was slightly protective for total coliform bacteria, and the presence of hog operations in zip codes was slightly protective for fecal coliform bacteria (Table 3). However, these findings were not significant for any type of operation with either indicator bacterium.

## 4. Discussion

Our data demonstrate that a majority of private wells included in this study are contaminated with fecal indicator bacteria and/or chemical contaminants at levels that exceed the SDWA drinking water quality guidelines set forth by the U.S. EPA. These findings are consistent with previous studies of private water wells that have been conducted in other states. A recent study of private wells in Pennsylvania found that 41% of wells failed to meet at least one drinking water standard [10], comparable with the 43% of wells that failed to meet one or more standards in our study. Similarly, in Wisconsin, an analysis of private water wells in rural areas found that 47% of these wells exceeded one or more health-based water quality standards [12]. Total coliform bacteria was also the most common microbiological contaminant in the Pennsylvania study and was detected in 33% of wells [10], comparable with the 25% of tested wells contaminated with total coliforms in our study. A recent study of private wells in Virginia found that 46% tested positive for total coliform bacteria, with 10% testing positive for *E. coli* [11]. Meanwhile, a North Carolina study of private wells found that 49% tested positive for total coliform bacteria and 6.4% tested positive for *E. coli* [14]. Previous studies have also indicated that seasonality may play a role in well water quality. [12,39]. In our study, the county with the highest percentage of wells that tested positive for fecal indicator bacteria was Kent County, which was sampled in the Fall (Table 1). However, because our study was cross-sectional, we did not collect samples over multiple seasons and, therefore, we cannot evaluate whether seasonal trends influenced our results. Nevertheless, our study adds to the growing body of research nationwide on the water quality of private wells that illustrates the need for improved monitoring of these wells.

Monitoring of fecal indicator bacteria in private well water is important for assessing the potential health risks associated with these water sources. To improve understanding of environmental factors that may impact private well water quality, we also investigated whether proximity to animal feeding operations was associated with microbial contamination of wells. Our data showed that there were no statistically significant associations between the presence of an animal feeding operation within a zip code and microbial contamination of private wells within the same zip code; however, this may be due to the small number of well water samples obtained during this initial study. Given that exposure to well water has been shown as an important risk factor for gastrointestinal illnesses [40,41,42], such as campylobacteriosis, exploration of this potential association deserves further study involving a larger number of private wells.

In a case-control study conducted in Sweden, Carrique-Mas et al. (2005) demonstrated that living in a household with a private well was a risk factor for *Campylobacter* infection (OR = 2.6; 95% CI = 0.9–7.4), although that association was not statistically significant [40]. Another case-control study conducted in Norway also found that the risk of campylobacteriosis was higher for those who obtained their water from a private household well compared with those receiving water from a public system (OR = 2.0; 95% CI = 1.2, 3.2) [41]. Consumption of water from a private well was also identified as a significant risk factor for sporadic campylobacteriosis (OR = 1.92; 95% CI = 1.46, 2.53) in a second Norwegian study by MacDonald et al. [42]. The potential for private wells to influence gastrointestinal illnesses such as campylobacteriosis (that are traditionally thought to be foodborne) remains understudied in the United States and deserves further attention.

One major challenge of improving private well water quality and reducing the risk of adverse health outcomes associated with this water source is that the numbers and locations of U.S. private wells are poorly characterized. Neither individual counties nor states have a complete database with addresses and other contact information for private well homeowners. As such, regular communications to homeowners reminding them to test their wells annually and delivering interventions where necessary is challenging. While the U.S. Geological Survey developed a nationwide inventory on the private well population [2], it was created using data on the population served by public water supply systems by county in each state and lacks the specific geographic locations of private wells. Creating a nationwide database of private well owners that is regularly updated by states could allow for improved evaluation of the factors that may influence well contamination, enhanced communication with well owners, and potential improvements in levels of waterborne illness.

In this study, we demonstrated the presence of fecal indicator bacteria in private drinking water wells in Maryland. As the presence of these indicator bacteria suggests a potential human health risk, well owners are often left to mitigate these risks through system repair, enhancement, or decontamination. However, knowledge of the contamination source of the well would be helpful in selecting an appropriate remediation method. Microbial source tracking (MST) is a collection of methods used to determine the likely source of contamination associated with the presence of fecal indicator bacteria [43]. MST has been previously used in a variety of applications, including in the management of surface water contamination and watershed remediation [43,44]. Allevi et al. (2013) utilized MST techniques to characterize the magnitude and incidence of microbial contamination in private wells in Virginia, and to identify the likely sources of this contamination [45]. Similarly, Krolik et al. (2014, 2016) analyzed well water samples from southeastern Ontario using MST to elucidate whether human or bovine sources were responsible for well contamination [46,47]. Future work relating to our study could include the application of MST methods to help identify the source of microbial contamination in Maryland wells, and to elucidate potential relationships between microbial contamination and environmental characteristics, particularly those relating to land use.

Given the small, cross-sectional nature of our study, there are several limitations to be considered. Our sample size of 118 households was relatively small, representing only a small fraction of the estimated 1,070,000 people who rely on private wells in Maryland [2]. Another limitation is the possibility that study participants may have improperly collected the water sample in their homes, which could then influence our ability to accurately determine their water quality parameters. We sought to minimize this potential problem by training participants on water sampling techniques during the safe drinking water clinic kickoff meetings, and by providing instruction sheets (along with the water sampling kits) on how participants should collect their water samples. Another limitation of this study is the use of U.S. Census of Agriculture data from 2007 with results from well water samples that were collected between 2013 and 2014. As noted above, the Census of Agriculture data were only available at the zip code level for the 2007 Census, and not for subsequent years. However, it is unlikely that the number of animal feeding operations in Maryland changed significantly between 2007 and 2013.

Despite these limitations, this is the first study to assess the water quality of private wells across multiple counties in Maryland, and to investigate the influence of animal feeding operations on well water quality, thereby addressing an important research gap in the state. This study also demonstrated the value in partnerships between land grant university research faculty and county-based extension faculty. Finally, the study highlighted the need for more educational outreach to private well owners in Maryland in order to improve private drinking water quality in the state. Additional studies are needed to identify and confirm potential factors that can influence private well water quality in Maryland, such as animal feeding operations, septic tanks, well construction characteristics, soil geology, and climatic conditions.

## 5. Conclusions

Our findings suggest that there are a significant number of private domestic wells in Maryland that do not meet the guidelines for well water quality set forth by the SDWA. This finding is similar to studies conducted in other states, including the nearby states of Virginia and Pennsylvania. In addition, while other studies have reported associations between proximity to animal feeding operations and microbial contamination of private wells, this association was not observed in this cross-sectional study and may have been influenced by our limited sample size. Further studies are needed to identify and confirm possible sources of contamination of private wells in Maryland. The lack of regular monitoring of private wells makes periodic assessments at national, regional, and local scales important sources of information about this key source of drinking water throughout the United States. The presence of microbial contaminants at levels greater than human health-based standards in 43.2% of private wells tested in this study highlights the importance of education and routine monitoring regarding the water quality of domestic wells to protect public health.

## Figures and Tables

**Table 1:** Dates on which the safe drinking water clinics were held.

Maryland County	Kick-Off Meeting	Interpretation Meeting
Cecil County I	March 2012	May 2012
Kent County	October 2012	December 2012
Montgomery	February 2013	March 2013
Cecil II	September 2013	November 2013
Queen Anne’s	February 2014	March 2014

**Table 2:** Characteristics of the safe drinking water clinic participants.

Characteristic	Category	Number (%) (*n* = 118)
County	Cecil (1)	25 (21.2)
	Kent	21 (17.8)
	Montgomery	25 (21.2)
	Cecil (2)	19 (16.1)
	Queen Anne’s	28 (23.7)
Age	18–49	17 (14.4)
	50–59	29 (24.6)
	60–69	40 (33.9)
	70–79	23 (19.5)
	≥80	9 (7.6)
Race/Ethnicity	African American	5 (4.2)
	Hispanic	1 (0.8)
	White	103 (87.3)
	Other or Unspecified	9 (7.6)
Level of formal education	<High school	1 (0.8)
	High School	10 (8.5)
	High school and some college	16 (13.6)
	Associate’s degree	9 (7.6)
	Bachelor’s degree	35 (29.7)
	Graduate degree	47 (39.8)
Number of years living at current home	1–10 years	34 (28.8)
	10–20 years	46 (39.0)
	More than 20 years	34 (28.8)
	Unknown	4 (3.4)
Previous testing of well water quality	Never	29 (24.6)
	Once	58 (49.2)
	Every few years	11 (9.3)
	Every year	4 (3.4)
	>Once per year	1 (0.8)
	Other or Unsure	12 (10.2)
Experienced diarrhea within the last 30 days	Yes	14 (11.9)
	No	104 (88.1)
Experienced vomiting within the last 30 days	Yes	0 (0%)
	No	118 (100%)

**Table 3:** Zip code-level analysis of the association between the presence of animal feeding operations and the occurrence of total and fecal coliforms in tested wells.

	Total Coliforms	Fecal Coliforms
Zip Code Variable	Odds Ratio (95% CI)	Odds Ratio (95% CI)
Cattle operations	1.23 (0.89, 1.68)	1.19 (0.82, 1.73)
Broiler operations	0.93 (0.84, 1.03)	1.10 (0.41, 3.00)
Hog operations	0.76 (0.49, 1.17)	0.81 (0.48, 1.37)
Dairy operations	1.12 (0.96, 1.31)	1.11 (0.93, 1.33)
Turkey operations	0.92 (0.68, 1.24)	1.24 (0.44, 3.47)
Aquaculture operations	1.32 (0.59, 2.93)	1.33 (0.52, 3.40)
